# Lipids that directly regulate innate immune signal transduction

**DOI:** 10.1177/1753425919852695

**Published:** 2019-06-10

**Authors:** Katherine C Barnett, Jonathan C Kagan

**Affiliations:** Harvard Medical School and Division of Gastroenterology, Boston Children’s Hospital, USA

**Keywords:** TLRs, innate immunity, myddosome, localization, phosphoinositides, PRRs, sorting adaptor, SMOC, cGAS, TIRAP

## Abstract

Pattern Recognition Receptors (PRRs) detect evidence of infection and tissue damage. The activation of these receptors and their downstream signal transduction pathways initiate a protective immune response. These signaling pathways are influenced by their spatial context, and precise subcellular positioning of proteins and protein complexes in these pathways is essential for effective immune responses *in vivo*. This organization is not limited to transmembrane proteins that reside in specific organelles, but also to proteins that engage membrane lipid head groups for proper positioning. In this review, we focus on the role of cell membranes and protein–lipid interactions in innate immune signal transduction and how their mechanisms of localization regulate the immune response. We will discuss how lipids spatially regulate the sensing of damage or infection, mediate effector activity, and serve as messengers of cell death and tissue damage.

## Introduction

Multiple signal transduction pathways operate in the innate immune system to link microbial detection to the initiation of host defense mechanisms. Mutations within key nodes in these pathways often result in life-threatening risks to the host, either through a loss of the ability to fight infections^1–3^ or through the generation of autoimmunity.^4^ Because of such a paramount role in the overall immune response, pharmaceutical approaches that boost or dampen these signaling pathways have demonstrable therapeutic potential.^5^ Therefore, defining the components of these pathways and their regulation is essential for understanding the overall immune response, identifying potential druggable targets, and implementing new strategies for disease intervention.

Innate immune cell signaling pathways have several common features that define their activity. First and most notably, these pathways generate immune responses to molecules generated during infection. At the apex of these pathways are germline-encoded protein sensors, known as PRRs, which bind to molecular motifs common to microbes (pathogen associated molecular patterns (PAMPs)) or molecules produced during events of damage (damage associated molecular patterns; DAMPs).^3^ Upon ligand recognition, these sensors activate intracellular signaling cascades to initiate host defenses. These defenses operate, in large part, through the up-regulation of genes encoding inflammatory mediators, such as cytokines and chemokines, or genes involved in cell-intrinsic defenses, such as interferons (IFNs) and IFN-stimulated genes.^3^ Second, receptor activation through ligand binding leads to receptor oligomerization and the subsequent formation of large multiprotein signaling platforms, known as supramolecular organizing centers (SMOCs).^6^ This conclusion is supported by extensive analysis of PRR families, such as Toll-like receptors (TLRs),^7–9^ RIG-I-like-receptors (RLRs),^10–12^ nucleotide binding leucine rich repeat containing proteins (NLRs),^13,14^ and the PRR cGAS.^15,16^ A third shared feature of these pathways is the regulation of signaling through spatial organization of PRRs and their downstream effectors. All aspects of innate immune signaling from ligand recognition to defense effector activities are controlled through specific localization, and the location of PAMPs and DAMPs similarly determines the type of immune response generated. Together, these features point to a unique set of signaling pathways that are optimized to respond rapidly to infection.

In this review, we discuss how spatial organization orchestrates these pathways, with a particular focus on the role of membrane lipids. Cell membranes may be viewed as barriers that serve a vital and singular function in compartmentalization of the cell, in which only transmembrane proteins are influenced through positioning on cell membranes. However, this simplified view does not account for the activities of peripheral membrane proteins, where electrostatic interactions with membrane lipids are critical for their localization and functions.^17–19^ These lipid-mediated activities are essential for the regulation and responses generated by PRR pathways and are a central focus of the discussion below.

## Spatial regulation of PRR activation and signal transduction

PRRs are positioned within cells to maximize rapid responses to microbial encounters. Such positioning places the host cell at a kinetic advantage by enabling detection of infection at its onset. For example, TLRs that detect bacterial or fungal cell surface components—such as TLR2, TLR4, and TLR5—are present at the cell surface.^3^ This positioning ensures microbial detection in the extracellular space. Microbial nucleic acids, in contrast, are rarely displayed on the surface of a potential pathogen. Consequently, the nucleic acid sensing PRRs, which include TLR3, TLR7–9, and murine TLR13, the RLRs and cGAS, are most commonly found within the cell, in either endosomes or the cytosol.^3^ These receptors are therefore poised to detect nucleic acids after microbial degradation in lysosomes or after viral uncoating in the cytosol. Furthermore, PRRs linked to inflammasome activation are located within the host cytosol to detect infection rapidly and also initiate pyroptotic cell death.

The loss of proper PRR localization can have catastrophic consequences for the host organism. For example, TLR9 transits through the secretory pathway in an inactive form to early and late endosomes, where proteolytic cleavage enables its ability to sense unmethylated CpG-containing DNA and initiate an inflammatory response.^20,21^ Altering the localization of TLR9 such that this protein is directed to the cell surface causes an autoinflammatory response in mice through the detection of extracellular self-DNA.^22^ Therefore, the specific location of these receptors is fundamental for self–nonself discrimination of DNA.

**Figure 1.**
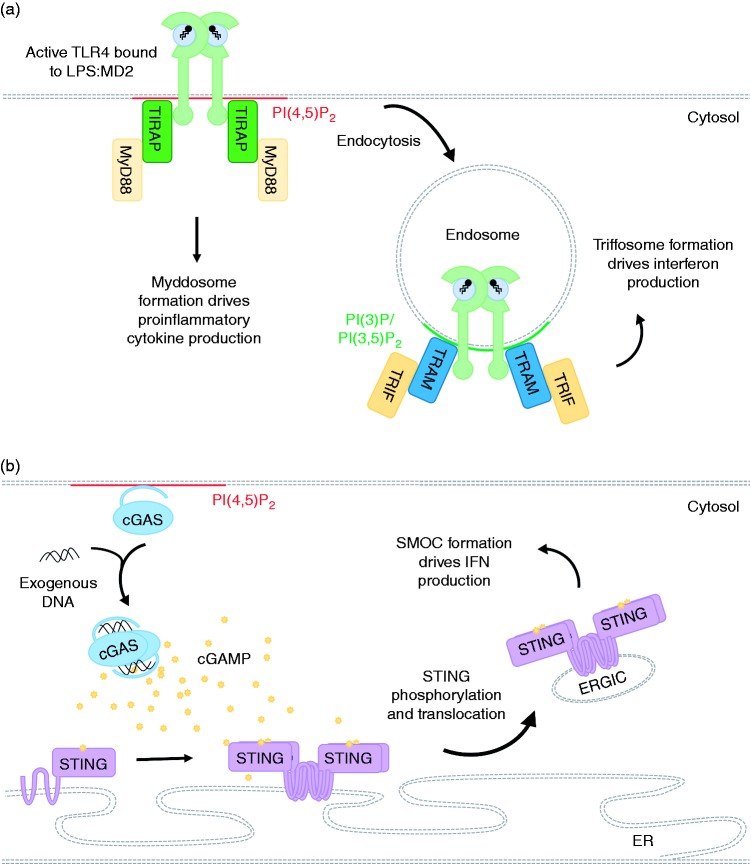
Subcellular localization directs supramolecular organizing center (SMOC) assembly and the response to innate immune stimuli. (a) TLR4 signaling outcomes are determined by its positioning and interaction with localization-dependent sorting adaptors. Active, TLR4 homodimers localize to phosphatidylinositol-4,5-bisphosphate (PI(4,5)P_2_)-enriched plasma membrane lipid rafts to interact with TIRAP to form the myddosome. Upon endocytosis, TLR4 interacts with TRAM on endosomes to form the triffosome. (b) RLR activation leads to SMOC formation through RLR oligomer-mediated MAVS aggregation. Shown is RIG-I mediated MAVS aggregation. Both peroxisomes and the mitochondria-associated membranes of the endoplasmic reticulum (ER) also serve as platforms for MAVS SMOC formation. Sensing of DNA by cGAS stimulates the production of cGAMP, which is bound by the ER resident protein STING. STING–cGAMP interactions leads to STING SMOC formation through its oligomerization, phosphorylation, and translocation to the ER-Golgi intermediate compartment to stimulate the synthesis of IFN.

Cell biological analysis of PRR activities has revealed an increasing number of examples of receptors whose subcellular sites of microbial detection are distinct from the sites of signal transduction. Indeed, it is now recognized that a necessary step in inflammatory signaling pathway activation by PRRs is the movement of ligand-bound receptors to a signaling-permissive subcellular location. Examples of this principle came first from the studies of TLR4. Upon binding LPS, TLR4 must first move into plasma membrane subdomains known as lipid rafts in order to drive inflammatory responses (Figure 1a).^23–25^ Subsequent movement of TLR4 into endosomes is necessary to maximize expression of these inflammatory genes and to induce the additional expression of IFNs,^18^ which drive NK cell activation and T cell–mediated adaptive immunity.^3^ Similarly, plasma membrane localized TLR2 must move into endosomes after microbial detection to promote maximal inflammatory gene expression,^26^ while TLR7 and TLR9 must move between endosomes after nucleic acid detection to stimulate inflammatory cytokine and IFN expression.^27,28^ In the cytosol, the RLRs RIG-I and MDA5 can presumably bind viral RNA in any location, but inflammatory and IFN responses only occur after their transport to the adaptor MAVS at the mitochondria, peroxisomes, or mitochondria-associated membranes (MAM) of the endoplasmic reticulum (ER).^29–31^ Finally, cGAS, which detects cytosolic DNA, produces 2′3′ cyclic GMP-AMP (cGAMP) upon DNA detection, and this secondary messenger must translocate to the ER, where it is detected by the protein STING.^32^ Only through activation of STING and its subsequent translocation from the ER can inflammatory and antiviral transcriptional responses be induced.^33,34^ Therefore, a common feature of these diverse pathways is the spatial dissociation of microbial sensing and initiation of pro-inflammatory signaling cascades, which could aid in the prevention of aberrant immune activation.

At the precise subcellular site of PRR signal transduction are proteins known as sorting adaptors, which are the only known factors that are present at the sites of signaling before any microbial encounter has occurred.^35^ As such, these proteins serve as landmarks of where in the cell signal transduction will eventually occur. The sorting adaptors TIRAP and TRAM operate in the TLR pathways, while the transmembrane proteins MAVS and STING operate in this manner in the RLR and cGAS pathways, respectively (Figure 1). These proteins must be engaged, either directly or indirectly, by upstream receptors in order to stimulate inflammatory and defensive gene expression. Mechanistically, sorting adaptors function as intracellular sensors of ligand-bound receptors. When ligand-bound receptors or the secondary messenger cGAMP enter the subcellular site of sorting adaptor residence, these adaptors stimulate the assembly of large multiprotein complexes known as SMOCs.^6^ SMOCs represent the signaling organelles of the innate immune system, operating as the principal subcellular site from which defensive signals emanate. Each sorting adaptor operates to seed the assembly of a different SMOC. For example, the sorting adaptor TIRAP, present on plasma membrane lipid rafts and endosomal membranes, serves to link most TLRs to the assembly of a SMOC known as the myddosome, which drives inflammatory gene expression (Figure 1a).^17,36^ The endosome-localized sorting adaptor TRAM serves an analogous function for TLR4 specifically, where it links ligand-bound receptors to an inflammation-inducing SMOC known as the triffosome (Figure 1a).^18^ In a similar manner, activated RLRs stimulate SMOC assembly by the MAVS adaptor on mitochondria, peroxisomes, and the MAM to drive inflammatory and antiviral responses.^29–31^ Furthermore, MAVS aggregation and SMOC assembly is also implicated in the induction of pyroptosis.^37^ Similarly, upon cGAMP binding, the adaptor protein STING forms a multimeric complex to activate antiviral and inflammatory gene expression (Figure 1b).^33,38,39^

The identification of sorting adaptors as molecular links between activated receptors and SMOC assembly explains the precise means by which PRR signaling is activated from discrete locations in the cell. Indeed, studies of the adaptors TIRAP, TRAM, and MAVS demonstrated that elimination of a sorting adaptor from its native subcellular position prevents proper signal transduction and inflammatory gene expression.^17,18,31^ In the next section, we will discuss recent studies of the means by which these sorting adaptors are regulated with particular focus on electrostatic protein–lipid interactions. This discussion will then be expanded to include other regulators of innate immunity that also use protein–lipid interactions for subcellular positioning with functional consequences for the host.

## Protein–lipid interactions as a mechanism of sorting adaptor positioning

Because SMOC assembly occurs at discrete locations in the cell, understanding the mechanisms that direct sorting adaptor localization to seed these complexes is an active area of research. Some of these mechanisms are self-evident, as the transmembrane proteins STING and MAVS are physically inserted into the membranes of eventual SMOC assembly. However, other sorting adaptors, which lack transmembrane domains, do not have a readily apparent mechanism of membrane association. In this section, mechanisms of membrane association that do not rely on transmembrane domains will be discussed, along with the specific mechanisms of membrane association by the sorting adaptors TIRAP, TRAM, and the *Drosophila* protein dMyD88.

Cell membranes are lipid bilayers comprised of amphiphilic phospholipids with hydrophobic lipid tails facing the inside of the membrane and hydrophilic head groups facing the extracellular space or the cytosol. Many proteins are able to interact electrostatically with the hydrophilic head groups that make up the membrane surface. Of the phospholipids that comprise cell membranes, the phosphoinositide phosphates (PIPs) play an important role in defining membrane spaces and in directing proteins to specific locations within the cell.^40^ PIPs are a dynamic group of membrane component lipids that are defined by specific phosphorylation modifications on the 3′, 4′, or 5′ carbons of a phosphatidylinositol head group.^40^ Several kinases and phosphatases regulate the phosphorylation patterns of these lipids, and they are rapidly converted as organelles change identity, such as early endosome transitioning to a late endosome.^40^ A well-characterized example of these lipids is phosphatidylinositol-4,5-bisphosphate (PI(4,5)P_2_), which is enriched on the plasma membrane. PI(4,5)P_2_ recruits several peripheral membrane proteins to the cytosolic face of the plasma membrane, such as phospholipase Cδ1, AP-2, and several actin-binding proteins.^41–43^ Interactions of these proteins with PI(4,5)P_2_ influences cell activities, such as endocytosis and phagocytosis.^42–45^ Other PIPs play similar roles on different organelles, such as PI(3)P on the cytosolic face of early endosomes^46,47^ or PI(3,5)P_2_ on late endosomes.^40,48^ Together, these lipids orchestrate many activities of peripheral membrane proteins through electrostatic interactions, and their activities and regulation are the subject of several recent reviews.^40,49–51^ In addition to PIPs, other membrane component lipid head groups can serve as binding partners for peripheral membrane proteins, such as phosphatidylserine (PS).^52^ Another mechanism of peripheral membrane association is through the post-translational addition of a lipid anchor, such as myristoylation, palmitoylation, or prenylation.^53^ These small lipid anchors can insert into cell membranes and interact with hydrophobic center of the cell membrane. Both of these mechanisms of membrane association are important for directing innate immune sorting adaptors and play essential roles in SMOC formation, which will be discussed in detail below.

The sorting adaptor TIRAP acts as a sensor of activated TLRs to form the myddosome, eliciting the expression of pro-inflammatory genes (Figure 1a). Prior to TLR activation, TIRAP is positioned on lipid raft microdomains within the plasma membrane through an interaction with PI(4,5)P_2_.^17^ TIRAP interacts with PI(4,5)P_2_ through a basic N-terminal phosphoinositide binding domain.^17^ Deletion of this domain leads to a loss of pro-inflammatory cytokine expression upon LPS treatment, preventing TLR4-mediated signaling, while reconstitution of TIRAP membrane association with another PI(4,5)P_2_ binding domain rescues this activity.^17^ However, although at steady state TIRAP is most concentrated at the plasma membrane, it is also capable of localizing to the endosomal compartments through interactions with other membrane lipids, namely PI(3)P, PS, and PI(3,5)P_2_.^36^ Like many PIP-binding domains, the N terminus of TIRAP is a promiscuous and intrinsically disordered PIP-binding domain. A recent study demonstrated that TIRAP interacts with multiple PIPs through a similar mechanism.^54^ This promiscuity of localization is key to TIRAP’s ability to serve as a sorting adaptor for both plasma membrane and endosomal TLRs. Indeed, TIRAP mediates myddosome formation upon LPS (TLR4-mediated) and CpG DNA (TLR9-mediated) stimulation.^36^ The promiscuity of TIRAP for multiple PIPs is important for its function, as mutant TIRAP proteins that display specificity for plasma membrane lipids are unable to mediate myddosome formation downstream from endosomal TLRs.^36^ Likewise, TIRAP mutants that display unique specificity for endosomal PIPs are unable to stimulate myddosome formation downstream from plasma membrane localized TLRs.^17^

The protein TRAM serves as another sorting adaptor for TLR signaling, mediating triffosome formation and the expression of type I IFNs upon TLR4 activation (Figure 1a).^55^ At steady state, TRAM localizes to the plasma membrane and endosomal compartments.^56^ The significance of plasma membrane localization is unclear, but its endosomal localization is necessary and sufficient to mediate IFN expression upon TLR4 activation by LPS.^18^ Unlike TIRAP, TRAM contains a bipartite membrane localization motif found in many different peripheral membrane proteins.^18^ The first seven amino acids of TRAM contain a myristoylation sequence, placing a lipid anchor on the protein’s N terminus.^56^ Directly adjacent to this motif is a short polybasic region that interacts promiscuously with PIPs and other acidic lipids.^18^ Together, these lipidation and lipid-binding motifs direct TRAM localization and function in the TLR4 pathway.

Similar to the TLR pathways found in mammals, the Toll pathway in insects is a critical regulator of antimicrobial immunity.^57^ The cell surface receptor Toll serves as a sensor of Gram-positive bacterial and fungal infections, with the sorting adaptor dMyD88 serving to initiate formation of a SMOC consisting of the adaptor Tube and the kinase Pelle.^58^ This SMOC induces the up-regulation of numerous NF-κB-dependent genes, most notably antimicrobial peptides (AMPs) that curtail infection.^57^ Similar to TIRAP in mammalian cells, dMyD88 localizes to the plasma membrane through an interaction with PI(4,5)P_2_,^19^ and this interaction is required for Toll-stimulated AMP production and surviving Gram-positive bacterial infections.^19^ Based on the similarities to mammalian TIRAP and TRAM, PIP-mediated membrane binding can be considered a conserved mechanism of TLR sorting adaptor activity in multicellular eukaryotes.

While PIPs position several TLR-associated sorting adaptors at sites of eventual signal transduction, there are also examples of PIP-mediated localization that dictate function after signaling initiation. These examples will be discussed below.

## Membrane lipids as mediators of innate immune effector activity

Innate immune signaling pathways follow a common sequence: pattern recognition nucleates SMOC formation, which activates inflammatory and defensive responses. Whereas many PRR pathways induce a host defense via the up-regulation of cytokines, chemokines, and IFNs, other pathways induce inflammation by processes of lytic cell death, namely pyroptosis and necroptosis.^59,60^ Like the transcription-inducing PRR pathways, specific localization of key signaling proteins within cells is essential for pyroptosis or necroptosis execution, and these activities are directed through protein–lipid interactions.

Recent studies have revealed an important role of the protein gasdermin D (GSDMD) in pyroptotic cell death.^61^ GSDMD exists in an autoinhibited state in the cell cytosol and is cleaved by cellular caspases upon inflammasome activation.^61^ Once cleaved, the N terminus of GSDMD interacts with membrane lipids to form pores in the plasma membrane, enabling the release of the inflammatory cytokine IL-1β and disrupting cellular ion gradients to facilitate cell death.^62–66^ *In vitro* binding assays demonstrated that GSDMD interacts with several lipids found on the inner leaflet of the plasma membrane, including PI(4)P, PI(4,5)P_2_, PS, and phosphatidylinositol (PI).^63,67^ Mutation of residues implicated in interactions with these membrane lipids prevented the GSDMD N terminus from associating with cell membranes and prevented GSDMD-mediated cell death.^63^ In addition, GSDMD has a high affinity for cardiolipin (CL), a lipid found in the inner mitochondrial and bacterial membranes.^68,69^ This binding activity for CL may allow GSDMD to kill *Escherichia coli* and *Staphylococcus aureus*, as they display CL on their cell wall.^63,67^

GSDMD is a member of a larger protein family collectively referred to as gasdermins (GSDMs).^70^ Like GSDMD, the N termini of almost all GSDM family members mediate cell death when overexpressed and are also capable of killing bacterial cells, and this includes GSDMA (murine GSDMA3), GSDMB, GSDMC, GSDMD, and GSDME (also known as DFNA5).^67^ Many of these family members bind PIPs *in vitro* and are implicated in pathologies linked to immune function, such as asthma and inflammatory bowel disease.^67,71^ Further research into this family of pore-forming proteins is necessary to understand the specifics of their activity and characterize better their interactions with membrane lipids in health and disease.

**Figure 2.**
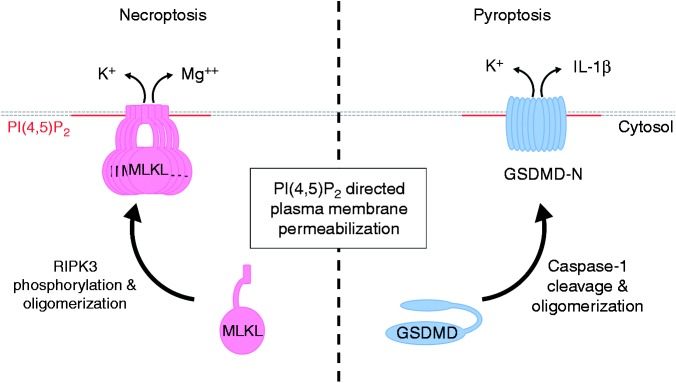
Phosphoinositide-directed membrane disruption is a common attribute of inflammatory cell death pathways. Both necroptosis and pyroptosis rely on plasma membrane pore formation to facilitate cell death. While the mechanisms of activation and the proteins mediating pore formation are unrelated, PI(4,5)P_2_ binding directs these pore-forming proteins to the plasma membrane.

In addition to the GSDM family, other pore-forming proteins mediate innate immune signaling through interactions with PIPs. For example, mixed lineage kinase domain-like protein (MLKL) forms pores to facilitate necroptosis.^72,73^ Upon phosphorylation by the necrotic executioner kinase RIPK3, MLKL oligomerizes and inserts into the plasma membrane to form a pore that disrupts ion gradients and leads to cell death.^72–74^ *In vitro* binding analysis of recombinant MLKL demonstrated that MLKL binds directly to PIPs, including PI(4)P and PI(4,5)P_2_.^75,76^ This activity is mediated by an N terminal helical bundle that contains several basic amino acids, which mutagenesis studies have implicated in plasma membrane recruitment of MLKL.^76^ Considering the similarities between MLKL and GSDMD, PIP-directed plasma membrane pore formation can be considered a common strategy of inflammatory cell death (Figure 2). Other examples of pore-mediated cell death in immunity include the extracellular proteins perforin and the complement membrane attack complex.^77,78^ However, these pore-forming proteins do not rely on specific phospholipids for their localization, and their mechanisms of targeting membranes and pore formation have been reviewed elsewhere.^77,78^

Membrane-directed innate immune effector activity is not limited to pore-forming proteins, as a recent study proposed that cytokine egress from the cytosol is mediated by membrane association.^79^ For example, the pro-inflammatory cytokine IL-1β localizes to the plasma membrane upon its cleavage by caspase-1.^79^ Mutation of a polybasic motif in IL-1β led to a significant decrease in its secretion from cells, and co-localization studies with the PLCδ1-PH domain suggest its membrane association may be mediated by PI(4,5)P_2_.^79^ However, direct interactions between PI(4,5)P_2_ and IL-1β have not been demonstrated. With these data, the authors proposed a model in which IL-1β maturation poises the cytokine for secretion through GSDMD pores and membrane blebbing. When considered with GSDMD and MLKL, these instances suggest that membrane positioning by PIPs is utilized for activities downstream of innate immune pathway activation and mediates further activation of the immune system after pathogen detection.

## Protein–membrane lipid interactions as a mechanism of PRR activation

Some of the best-characterized activators of innate immunity are lipids, such as the Gram-negative bacterial cell wall component LPS. However, recent research has detailed that endogenous lipids also serve as stimulators of innate immune signaling. Upon tissue injury and cell death, membrane phospholipids become spontaneously oxidized and are capable of mediating inflammation in the absence of infection.^80,81^ As such, these oxidized lipid molecules are DAMPs that are implicated in various disease states, including atherosclerosis and acute lung injury.^81–83^

Oxidized derivatives of 1-palmitoyl-2-arachidonoyl-*sn*-glycero-3-phosphorylcholine (PAPC), collectively referred to as oxPAPC, are one class of DAMPs generated through oxidation of membrane component phospholipids and upon tissue injury, can reach concentrations as high as 100 μM in the blood.^82,83^ oxPAPC binds the LPS receptor CD14, and CD14-mediated capture of oxPAPC allows for the internalization of these ligands and transport to the cell cytosol, where oxPAPC activates the inflammasome regulator caspase-11.^84,85^ Indeed, following priming with various TLR ligands, oxPAPC treatment of DCs led to the secretion of IL-1β and IL-18, cytokines only released from the cell upon inflammasome activation.^84^ *In vitro* binding assays and intracellular immunoprecipitation assays revealed that oxPAPC interacts directly with caspase-11 and caspase-1 to form the inflammasome.^84,85^ However, unlike other activators of inflammasome activity, such as intracellular LPS, ATP, or nigericin, oxPAPC-mediated inflammasome activation did not lead to pyroptotic cell death in addition to IL-1β release in DCs.^84^ Study of components of oxPAPC indicate that different oxidation products may have differential stimulatory capacity in various cell types.^85–88^ For instance, the oxPAPC component molecules 1-pamitoyl-2-glutaryl-*sn*-glycero-3-phosphocholine (PGPC) and 1-palmitoyl-2-(5’-oxo-valeroyl)-*sn*-glycero-3-phosphocholine (POVPC) induce inflammasome activation and IL-1β secretion from living (hyperactive) DCs and macrophages.^85–87^

While oxPAPC acts in the aforementioned manner as an inducer of inflammation, its actions are context dependent. For example, oxPAPC pre-treatment of naïve cells has long been known to prevent subsequent responses to LPS,^89^ probably due to the fact that both of these lipids interact with the same amino acids in CD14.^85^ These conditions may represent sterile tissue injury, where oxPAPC serves to prevent detection of potential endogenous TLR4 ligands. In contrast, the pro-inflammatory activities of oxPAPC and LPS co-detection may represent events that occur at sites of pathogen interactions, where PAMPs and DAMPs are commonly found. In these ways, membrane-derived oxPAPC serves as either an activator or inhibitor of innate immune signaling, depending on the context in which this lipid is encountered.

Finally, recent work suggests that lipid interactions play an important role in regulating PRR activity within the cytosol. First, a recent study of NLRP3, a regulator of inflammasome activity, determined that this protein localizes to the trans Golgi network (TGN) upon various stimuli.^90^ Binding to PI(4)P was proposed as the mechanism of TGN association by NLRP3, and amino-acid residues implicated in PI(4)P binding were required for NLRP3-mediated inflammasome assembly and activation.^90^ Previous work described NLRP3 association with the mitochondria through an interaction with cardiolipin.^91,92^ Further research will clarify these discrepancies and pinpoint the specific localization of NLRP3.

Another example of PRR regulation through PIP binding comes from a recent study of the intracellular DNA sensor cGAS, which demonstrated that inactive cGAS associates with the plasma membrane through an interaction with PI(4,5)P_2_ (Figure 1b).^93^ This activity was mediated by an N-terminal localization domain that binds PI(4,5)P_2_.^93^ Loss of this domain resulted in cGAS hypersensitivity to self-DNA that could be rescued by the addition of a known PI(4,5)P_2_ binding domain.^93^ Thus, PIPs mediate innate immune signaling activity at the level of the receptor, the effector, and perhaps also the cytokines whose activity they release.

## Perspectives

Based on the ample evidence that cell membranes and their component PIPs operate at various stages in innate immune signaling networks, several questions arise. For example, almost all of our knowledge of lipid-binding proteins in the innate immune system is derived from studies of pathway activation. Whether analogous systems operate at later stages of the signaling response, potentially as mechanism of pathway down-regulation, is less clear. One potential protein with such activity is the TLR regulatory factor Tollip.^94^ Tollip contains a C2 domain that interacts with several PIPs *in vitro* in a calcium-independent manner^95,96^ and localizes to endosomes where it interacts with several myddosome components.^94,97,98^ Genetic analysis suggests Tollip operates as a negative regulator of TLR signaling, but the mechanism of this regulation remains unclear.^97^ Furthermore, pathways in the innate immune system are also likely subject to regulation by lipid-binding factors, as an increasing body of literature has implicated PIP kinases and phosphatases in immune signaling. For example, these proteins regulate the localization and function of TIRAP in the TLR pathway,^99^ thus providing an example of direct regulation of signaling by lipid-based protein localization. Likewise, due to their central role in vesicle trafficking and endocytosis,^40^ these enzymes likely have indirect roles in PRR pathway activation by enhancing or limiting access of endosomal or cytosolic PRRs to PAMPs and DAMPs. Consistent with this idea, autophagy pathways that deliver cytosolic viruses to endosomal TLRs are sensitive to PI3K inhibitors.^100^ Together, these few examples provide context for a broader discussion of how lipid-binding proteins influence PRR pathways in many ways, but also raise the question of how much we do not know.

Given the complexity of pathogen and damage sensing, the organization and regulation of innate immune signaling pathways by cell membranes and their component lipids cannot be overlooked. Membranes are fundamental to the cell itself, defining its periphery and compartments and acting as gatekeepers to the extracellular world. Within the context of innate immunity, membranes and their component lipids define the difference between self and nonself, as exemplified by TLR9, serve as platforms for pathway activation, and direct the downstream activity of these pathways to activate other components of the immune system. These membranes are even capable of activating the immune system, as cell death leads to the production of oxPAPC. However, these are singular examples in a wide network of signaling pathways. To understand innate immunity better is to understand the cell biology better that serves as its context. We expect much future research will be focused on better defining the role of lipids and the proteins they interact with as central regulators of immunity and defense.
